# Perseus: perception with semantic endoscopic understanding and SLAM

**DOI:** 10.1007/s11548-026-03717-w

**Published:** 2026-05-26

**Authors:** Ayberk Acar, Fangjie Li, Susheela Sharma Stern, Lidia Al-Zogbi, Hao Li, Kanyifeechukwu Jane Oguine, Dilara Isik, Brendan Burkhart, Jesse F. d’Almeida, Robert J. Webster, Ipek Oguz, Jie Ying Wu

**Affiliations:** 1 Department of Computer Science, Vanderbilt University, 2301 Vanderbilt Place, Nashville, TN 37235 USA; 2 Department of Mechanical Engineering, Vanderbilt University, 2301 Vanderbilt Place, Nashville, TN 37235 USA; 3 Laboratory for Computational Sensing and Robotics, Johns Hopkins University, 3400 N Charles St., Baltimore, MD 21218 USA

**Keywords:** 3D Reconstruction, Endoscopy, Registration, Segmentation, Depth estimation, NOTES

## Abstract

**Purpose:**

Natural orifice surgeries minimize the need for incisions and reduce recovery time compared to open surgery; however, they require higher expertise due to visualization and orientation challenges. To enable reliable scene understanding for surgeon guidance and automation, we propose a perception pipeline that generates semantically informed 3D reconstructions.

**Methods:**

We bring learning-based segmentation, depth estimation, and 3D reconstruction modules together. Through segmentation, we delineate tumor and prostate lobe borders, and through depth estimation and real-time SLAM-based reconstruction, we generate dense 3D point clouds from monocular videos. By propagating 2D labels into 3D space, we create real-time segmented maps of the surgical scenes. Additionally, we use registration with robot poses to solve the scale ambiguity of mapping from monocular images and allow the use of semantically informed real-time reconstructions in robotic surgeries.

**Results:**

We achieve sub-millimeter reconstruction accuracy based on average one-sided Chamfer distances, average pose registration RMSE of 0.9 mm, and an estimated scale within 2% of ground truth. Compared to offline Structure-from-Motion baseline, the proposed SLAM-based approach improves processing time while maintaining or improving reconstruction accuracy. Qualitative evaluations show robustness in challenging scenarios, including submerged prostate experiments and cadaver airway explorations.

**Conclusion:**

We present a modular perception pipeline, integrating semantic segmentation with real-time monocular SLAM for natural orifice surgeries. This pipeline offers a promising solution for scene understanding that can facilitate automation or surgeon guidance. Due to its plug-and-play design and demonstrated generalizability across anatomies and experimental conditions, this framework provides a scalable foundation for future clinical translation.

## Introduction

With the advancements in minimally invasive surgery technologies, new approaches such as endoluminal, transluminal, and natural orifice transluminal endoscopic surgeries (NOTES) have gained popularity [1]. NOTES aim to minimize the number of incisions and use natural access points in the body. NOTES allow operating through natural orifices such as the mouth, urethra, anus, or vagina, without additional incisions; however, at the cost of increasing complexity. Difficulties in visualization, spatial orientation, depth perception, and anatomy recognition make these procedures challenging, throttling expertise and practical applicability [2]. These limitations show the significant need for better scene understanding to guide surgeons and automation.

We present a perception pipeline for NOTES that incorporates real-time 3D reconstruction from monocular images, depth estimation, and segmentation. In our previous work [3], we showed that offline monocular vision guidance can enable automated tumor removal. This work focuses on solving the limitations of the offline perception pipeline by creating a real-time solution to build segmented, scaled, and registered-to-surgical-scene 3D reconstructions of a surgical field. Furthermore, we extend the evaluation of 3D reconstruction to a different anatomy to demonstrate generalizability and comparatively evaluate mapping methods. Codebase for this project is available on GitHub: URL: github.com/vu-maple-lab/perseus

## Background and related work

**Clinical relevance: ** NOTES technique, originally developed for abdominal surgeries, later gained interest in different application areas such as thoracic or urologic surgeries [4, 5]. In this study, we focus on two clinical problems: central airway obstruction (CAO), and benign prostatic hyperplasia (BPH), which can be treated via oral and urethral access, respectively.

CAO is a disorder with increasing prevalence and is a cause of significant morbidity and mortality [6]. Therapeutic tools such as laser and electrocautery that are delivered through bronchoscopes can enable complete removal of the obstructing tumor [6], but the procedure is challenging and complications can be fatal [7, 8].

BPH refers to growth of prostate lobes and is very prevalent in aging males [9]. Lower urinary tract symptoms and bladder outlet obstruction (BOO) can stem from BPH, which can affect patients’ quality of life. BOO may interfere with sexual functioning and potentially cause urinary tract infections, formation of bladder stones, and renal failures [9]. Treatment options for BPH vary from medical therapy to surgical interventions such as transurethral resection of prostate.

Recent studies show that robotic systems can be used for CAO removal with reduced complications [8]. Additionally, with proper visual guidance, automation of this procedure is feasible, allowing precise and consistent performance. Previous studies show the feasibility of tumor removal on a benchtop setting with open surgery models [3, 10]. Autonomous features for robotic BPH treatment techniques, such as using a robotic arm to direct a high-pressure water jet for tissue removal, may improve outcomes [11]. Accurate mapping and segmentation for transurethral approaches can extend automation to other surgeries.

**3D reconstructions: ** In our previous work, we combined 3D reconstruction of monocular images with segmentation methods to guide the automation of tumor removal [3]. However, the Structure-from-Motion (SfM) method used in our previous study is time-consuming, and repeating the reconstructions in case of failure, or as the operation progresses, is not feasible.

Simultaneous Localization and Mapping (SLAM) algorithms offer a good alternative to SfM, allowing real-time reconstructions and camera pose estimations. Previous studies show the feasibility of SLAM applications in a wide range of laparoscopic and endoscopic surgeries such as sinus endoscopy, colonoscopy, and ureteroscopy [12–14]. Previous work demonstrated that integration of explicit depth estimation and priors can improve SLAM reconstruction results compared to using monocular video alone [13, 14]. In terms of segmentation integration into SLAM, literature mostly focused on outlier detection, use of semantic information to improve SLAM performance, or creating segmented scenes for natural images [15, 16]. Other work proposed the use of segmentation to remove dynamic tools in the surgical scenario, to reduce reconstruction errors [17]. However, literature focusing on use of semantic information to detect target anatomies and create informed surgical scenes is very limited. Methods such as Semantic-SuPer bring together information from depth estimation, semantic segmentation, and tool poses to create segmented surgical scenes [18]. This method is tested only on an open access laparoscopy setup and not implemented for endoscopy.

In this study, we combine the real-time 3D reconstructions with semantic target information acquired from a deep learning model. This allows fast and accurate scene understanding in endoscopic surgeries, enabling downstream tasks such as surgical automation. To the best of our knowledge, this study is the first to use real-time SLAM methods for CAO and BPH, creating new possibilities for surgical approaches.

**Fig. 1:**
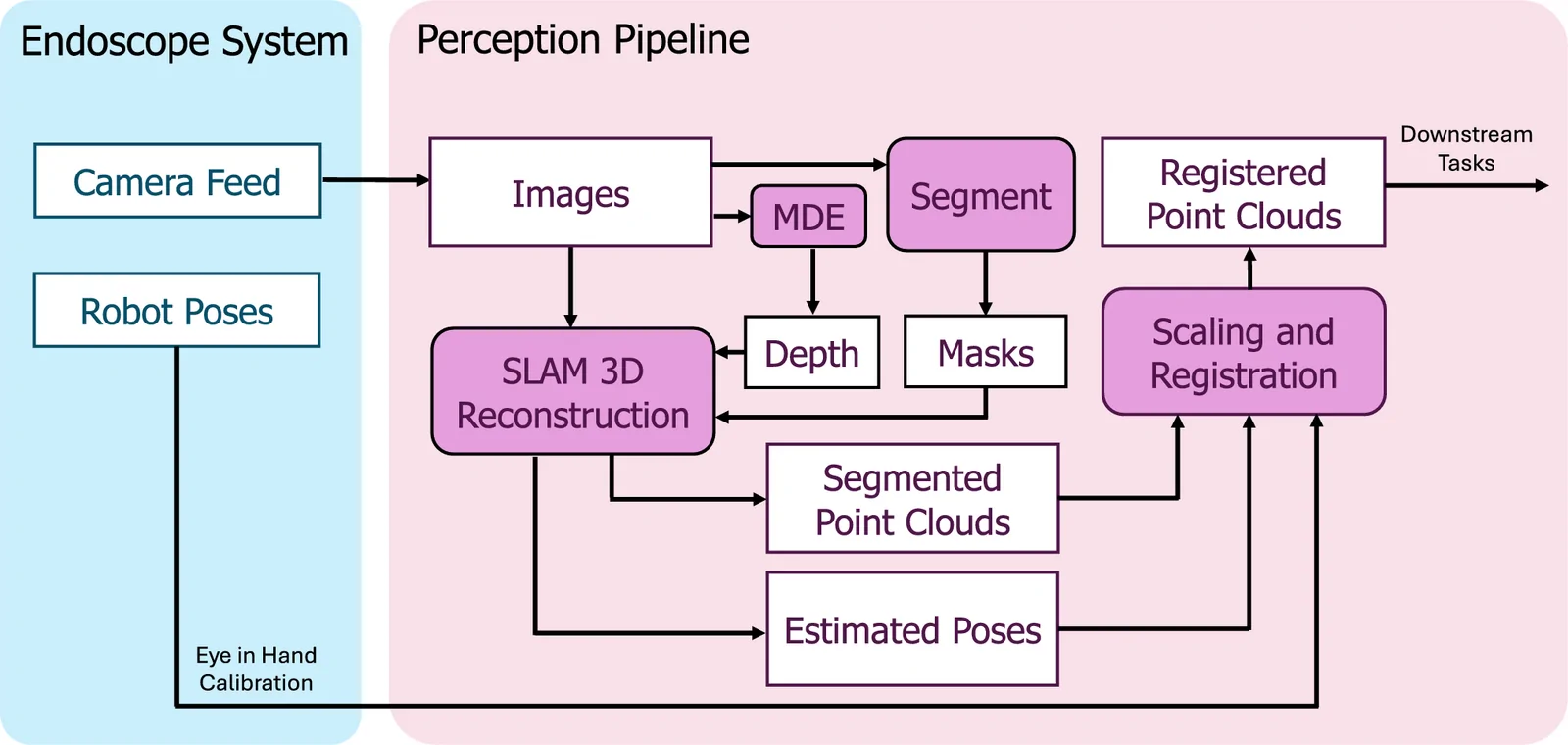
Diagram showing the overall workflow. We use depth estimations and target segmentation masks in the SLAM system to create segmented point clouds of the surgical scene. We use estimated and robot camera poses to scale and register these reconstructions, to be used in downstream tasks

## Methods

An overall workflow can be seen in Fig. 1. This section details the modules of our workflow and the data collection. Our pipeline predicts segmentation masks and depth maps for each frame in the image stream, and creates segmented point clouds with a modified reconstruction algorithm. After the reconstruction, these point clouds can be used directly as a visual guidance, or can be scaled and registered with a registration algorithm that uses robot kinematics and estimated camera poses. We explain the reconstruction algorithm in detail in subsection Real-time 3D reconstruction, segmentation and monocular depth estimation (MDE) model in Segmentation and monocular depth estimation, segmented point cloud generation in Segmented reconstructions and scaling and registration in Registration and scaling the reconstruction.

### Phantom production and data collection

**Fig. 2:**
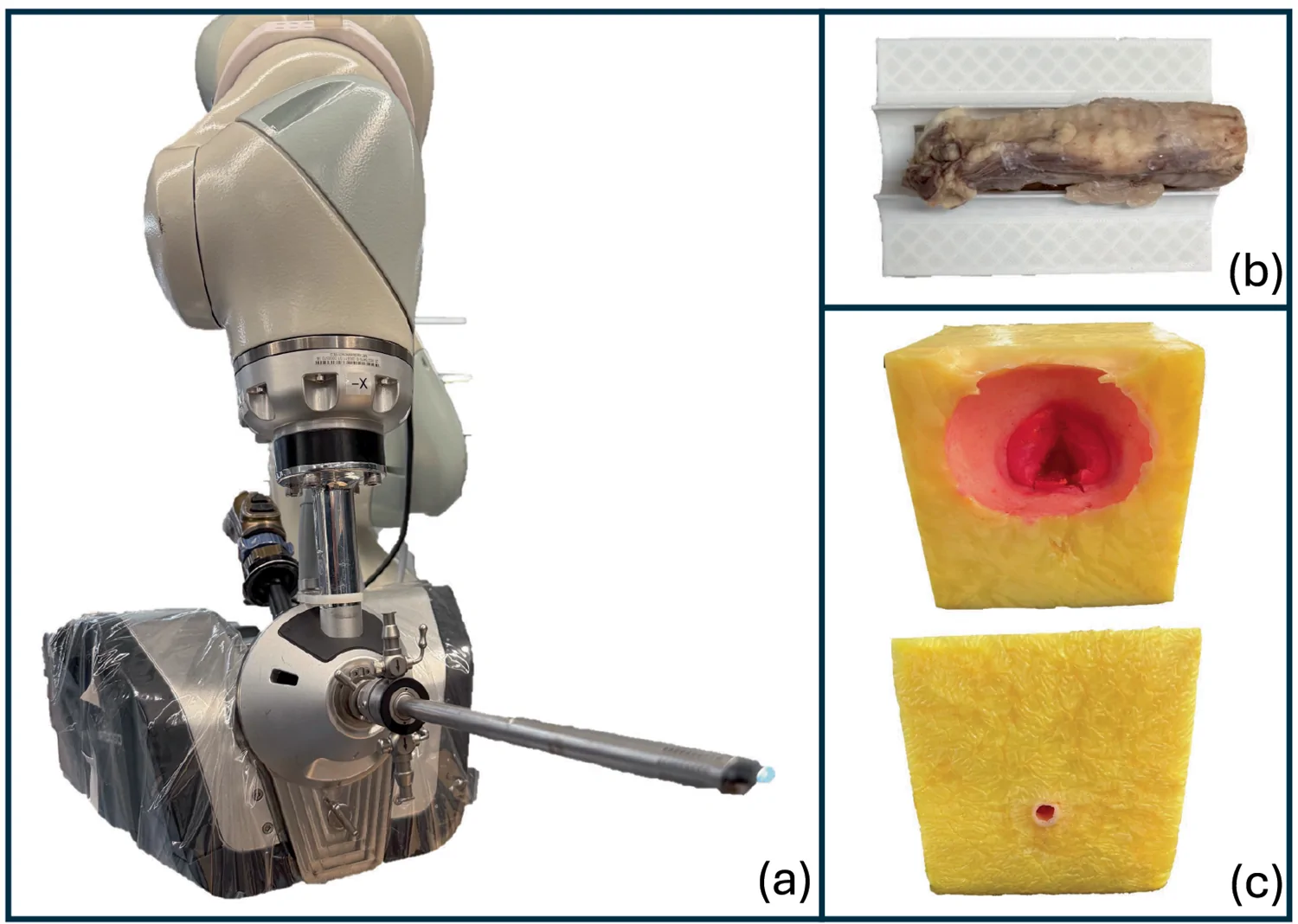
Camera system and phantoms used. Endoscope held by robotic arm **(a)** is inserted to trachea phantom from the end points **(b)** and prostate phantom from the urethra **(c)** to collect videos

For the CAO case, following our previous work [3, 19], we prepare phantoms using sheep pluck and chicken breast to mimic human anatomy. We separate the trachea from the rest of the pluck for easier handling. We cut small pieces of chicken breast that cause around 50% occlusion in the trachea, place them inside the airway through small incisions, and secure them with super glue (Fig. 2b).

For our BPH experiments, we use hydrogel phantoms developed originally for surgical training [20]. These phantoms are modeled after patient CT scans and reflect the visual and material properties of human anatomy (Fig. 2c).

We record monocular videos on both phantoms with Virtuoso Surgical (Nashville, TN, USA) endoscopy system (Fig. 2a). Additionally, to acquire ground-truth geometries for evaluation of our perception pipeline, we take computed tomography (CT) scans of the phantoms and segment the anatomies manually.

### Segmentation and monocular depth estimation

We train two separate segmentation models for CAO tumor identification and BPH lobe differentiation. Although the models share an identical network architecture and training configuration, they differ in the dataset composition used for training.

Our segmentation framework builds upon a U-Net–style [21] architecture integrated with SAM2 [22] as the encoder backbone. To efficiently adapt SAM2 to the CAO domain, we employ adapter-based fine-tuning [23], where lightweight adapter modules are inserted into the transformer blocks to update task-specific parameters while keeping the majority of the pretrained weights frozen. The fine-tuning of SAM2 follows the approach outlined in our recent work [24], which provides comprehensive details on the model design, data preprocessing, objective function, and optimization strategy for CAO segmentation. The same architectural design and training strategy were applied to the BPH segmentation task. In this case, the model was trained on approximately 3,300 images, containing a diverse range of surgical actions on the phantoms, from exploratory views of the BPH anatomy to operative environments involving tool interactions and tissue deformation.

To address the lack of ground-truth depth in endoscopic images, we use an MDE framework [25] that integrates 3D CT data and 2D endoscopic video data. Our MDE consists of three main stages: (1) rendered data generation in Unity using 3D CT segmentations of CAO and BPH scans to produce absolute depth maps [26]; (2) unsupervised domain adaptation [27] between rendered and real endoscopy data, where rendered images are translated into the visual style of real endoscopic scenes to reduce the domain gap; and (3) depth prediction by fine-tuning the DepthAnythingV2 model [28] with paired translated rendered images and depth maps from step (2), achieving accurate depth estimation. Separate models were trained for CAO and BPH, respectively. Further analysis for segmentation [24] and MDE [25] models can be found in corresponding publications.

**Fig. 3:**
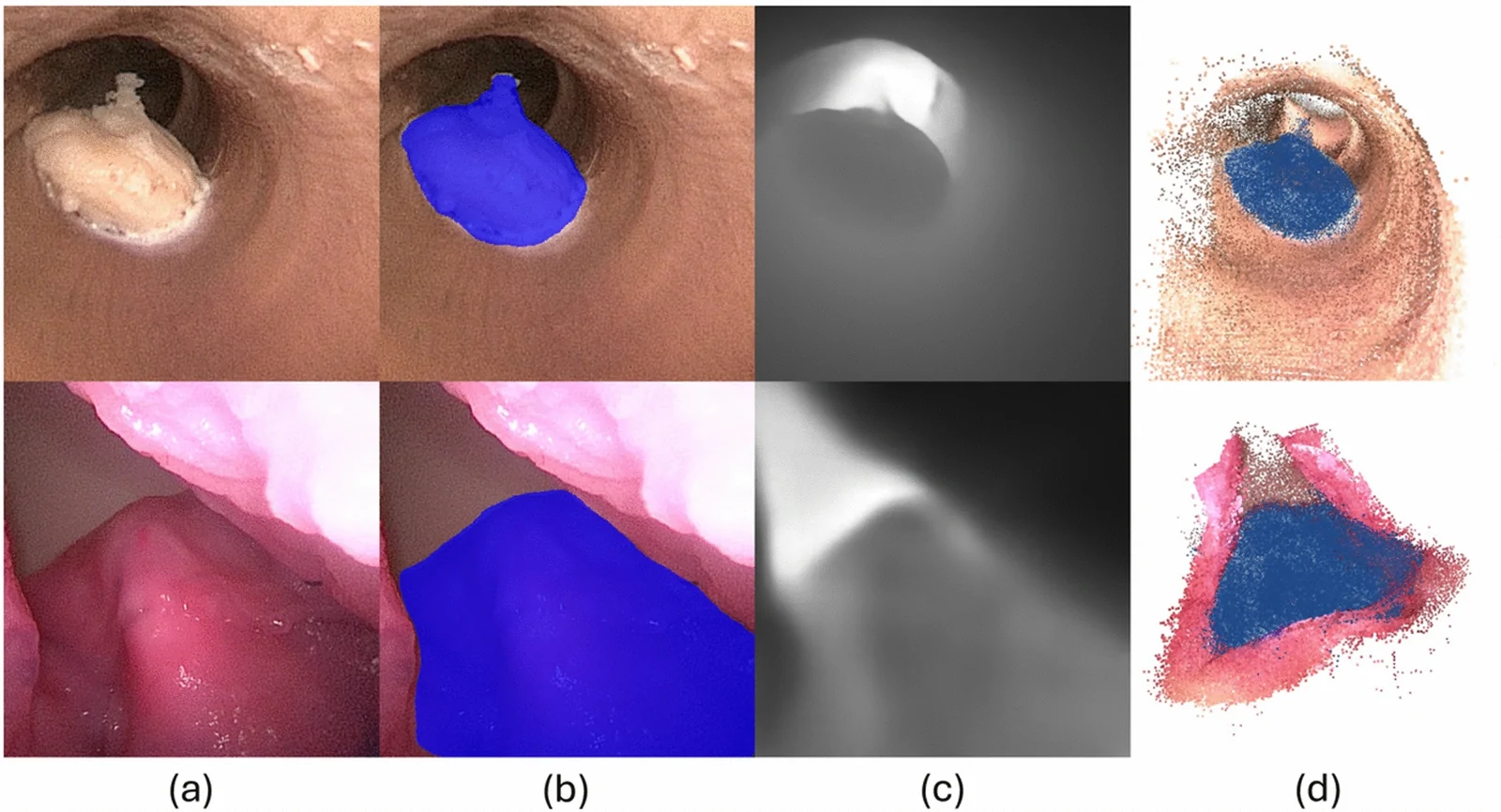
**(a)** Example endoscope images, **(b)** segmentation overlays, **(c)** monocular depth estimation results, **(d)** segmented 3D reconstructions. Top row shows central airway obstruction and bottom row shows the benign prostatic hyperplasia case

### Real-time 3D reconstruction

To create 3D reconstructions in real time, we use DROID-SLAM [29]. DROID-SLAM uses deep learning to iteratively update and correct estimated camera poses and predicted per-pixel depth values for the corresponding frames. The corrected depth values are later mapped to a point cloud in 3D using an inverse projection function and the pinhole camera model. DROID-SLAM supports multiple input modes, such as RGB-D or stereo. To augment our monocular video, we integrate our customized in-domain MDE model to create priors for the SLAM algorithm. This results in a pseudo-RGB-D pipeline (referred to as RGB-D SLAM), without the bulky and expensive depth cameras. We use the DROID-SLAM model weights from [29] without additional training, testing the generalization capability of the reconstruction algorithm.

We perform a checkerboard calibration before explorations to obtain the camera parameters required by the SLAM framework. Since the endoscope has a fisheye effect for an increased field of view, following the original DROID-SLAM implementation, we undistort the camera stream. We crop the images around the image center to remove the black mask around the endoscope view. Then, we scale these preprocessed images to match SLAM’s model input size. During the reconstruction, for the inverse projection, we use the estimated rectified camera matrix acquired from the camera calibration for the corresponding undistorted images.

Using DROID-SLAM with the monocular image stream from the endoscope allows us to create point clouds in real-time. However, these reconstructions do not have the crucial context information, such as tumor borders, required to identify the regions of interest for automation. We integrate a segmentation pipeline to the reconstruction process, to create semantically informed scene maps.

### Segmented reconstructions

To identify the regions associated with the targeted areas in the point cloud, we run a segmentation model inference in each frame in the image stream. At the inverse projection step of the reconstruction algorithm, we rescale the segmentation masks to match the size of the final inverse depth maps and use them to index the 3D point cloud acquired from depth images. Points corresponding to the pixels fall into the segmentation region are stored separately and visualized in a different color (Fig. 3d).

### Registration and scaling the reconstruction

Although we use an MDE model that outputs absolute depth estimation, imperfections in this model can still cause scale differences in the monocular reconstruction. While these reconstructions are still useful as a 3D map, correct scale is required for applications such as automation and measurements. To overcome the scale ambiguity and register the estimated map onto the surgical scene, we use robot kinematics data of the camera motion. By reading the robot arm poses and applying an eye-in-hand calibration [30], we acquire the robot-held-camera pose readings. We use the Umeyama [31] algorithm to register the translation components of estimated camera poses from SLAM (post-global bundle adjustment) to robot-held-camera pose readings. This registration step is performed offline after reconstruction ends and is used to estimate a single global scale factor (and rigid alignment) for the reconstructed map without affecting reconstruction quality. Registration of these two independently acquired poses avoids the fiducials required in our previous work [3] and allows immediate scaling and alignment of the segmented 3D maps to the physical scene. This method can be generalized to non-robotic applications by acquiring the camera poses using means such as optical trackers.

**Fig. 4:**
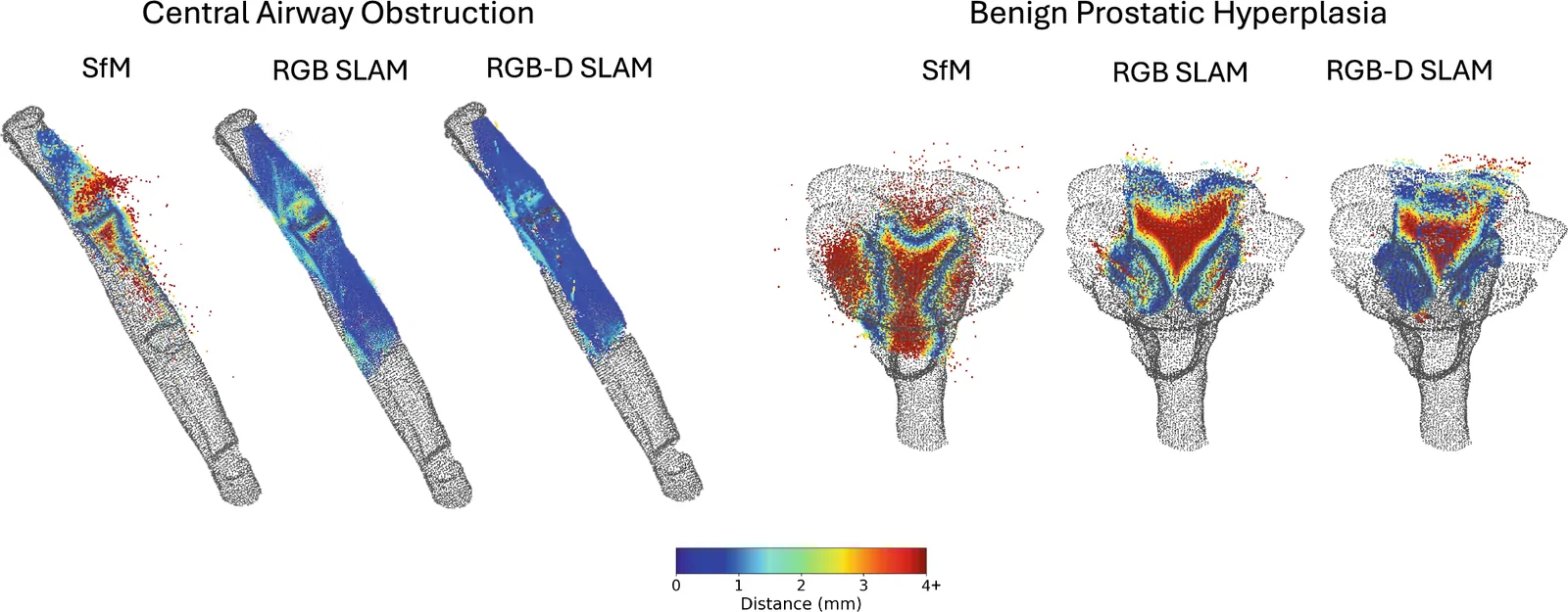
Example evaluation of reconstructions with registration to CT scan point cloud. Heatmaps indicate distance to the closest point

## Experiments and results

**Table 1:** Average quantitative evaluation results for 3D reconstruction methods. ↑ indicates higher is better, ↓ indicates lower is better. Best results in the category are given bold and the second best is underlined. SLAM processing times are reported before and after bundle adjustment (BA)

*Central airway obstruction*	**SfM**	**RGB SLAM**	**RGB-D SLAM**
Median closest point dist. (mm) ↓	0.63 ± 0.13	**0.52 **±** 0.23**	$\underline{0.53 \pm 0.32}$
One-sided Chamfer dist. (mm) ↓	0.90 ± 0.17	$\underline{0.67 \pm 0.30}$	**0**.**66** ±** 0.40**
One-sided Hausdorff dist. (mm) ↓	100.96 ± 127.29	$\underline{15.10 \pm 5.27}$	**6.92 **±** 2.24**
Pre-BA time per frame (sec) ↓	-	**0.26 **±** 0.02**	$\underline{0.29 \pm 0.02}$
Post-BA time per frame (sec) ↓	6.39 ± 1.98	**0.42 **±** 0.07**	$\underline{0.46 \pm 0.07}$
Segmentation precision (%) ↑	**96.69 **±** 1.59**	85.48 ± 5.09	$\underline{88.07 \pm 2.56}$
# Reconstruct. Points (x1000) ↑	121.6 ± 44.6	**1021.2 **±** 259.3**	$\underline{588.1 \pm 130.0}$
Coverage (%) ↑	27.76 ± 11.97	**43.84 **±** 5.97**	$\underline{35.55 \pm 6.14}$
*Benign prostatic hyperplasia*	**SfM**	**RGB SLAM**	**RGB-D SLAM**
Median closest point dist. (mm) ↓	1.86 ± 0.30	$\underline{0.63 \pm 0.19}$	**0.52 **±** 0.15**
One-sided Chamfer dist. (mm) ↓	2.25 ± 0.40	$\underline{0.89 \pm 0.19}$	**0**.**65** ±** 0.19**
One-sided Hausdorff dist. (mm) ↓	31.63 ± 13.95	$\underline{21.25 \pm 14.61}$	**9.95 **±** 1.78**
Pre-BA time per frame (sec) ↓	-	**0.21 **±** 0.05**	$\underline{0.23 \pm 0.05}$
Post-BA time per frame (sec) ↓	16.76 ± 8.70	**0.26 **±** 0.05**	$\underline{0.28 \pm 0.05}$
Segmentation precision (%) ↑	**96.23 **±** 2.64**	89.71 ± 5.18	$\underline{90.87 \pm 5.40}$
# Reconstruct. points (x1000) ↑	84.3 ± 50.7	**467.5 **±** 236.3**	$\underline{288.3 \pm 134.6}$
Coverage (%) ↑	$\underline{20.85 \pm 2.53}$	**23.52 **±** 4.21**	20.13 ± 2.65

### Reconstruction quality

We comparatively and quantitatively evaluate the reconstruction accuracy and the segmentation precision on five exploration videos acquired from three different CAO phantoms and two exploration videos acquired from two different BPH phantoms. Extracted frame numbers vary from 512 to 1114. To quantify the reconstruction accuracy independently from other components, we scale and register the 3D reconstructions to the segmented ground-truth CT point cloud manually using visual cues. We initialize the position and scale of the reconstructions and fine-tune the registration with Iterative Closest Point (ICP) algorithm (Fig. 4).

Our baseline for comparison is the DISK [32]+LightGlue [33] SfM pipeline of the Hierarchical Localization Toolkit [34], used in our previous work [3]. This time, for a fair comparison, we also undistorted the images and center-cropped the endoscope mask same way as the SLAM pipeline input before feeding into the SfM algorithm. We also compare the performance of SLAM with and without the MDE integration.

Table 1 shows the quantitative results. SLAM algorithm results are post-global bundle adjustment. Coverage means the percentage of CT point cloud points that have a correspondence in reconstruction, within 1-mm distance. The one-sided Chamfer and Hausdorff distances are calculated from reconstruction to CT scan models. Figure 4 shows qualitative evaluations of one sequence for each phantom case, for the closest point metric. Experiments are done on an NVIDIA RTX 4090 GPU. During our real-time experiments, we do not observe a significant delay before the global bundle adjustment step that takes place at the end of the image stream.

**Fig. 5:**
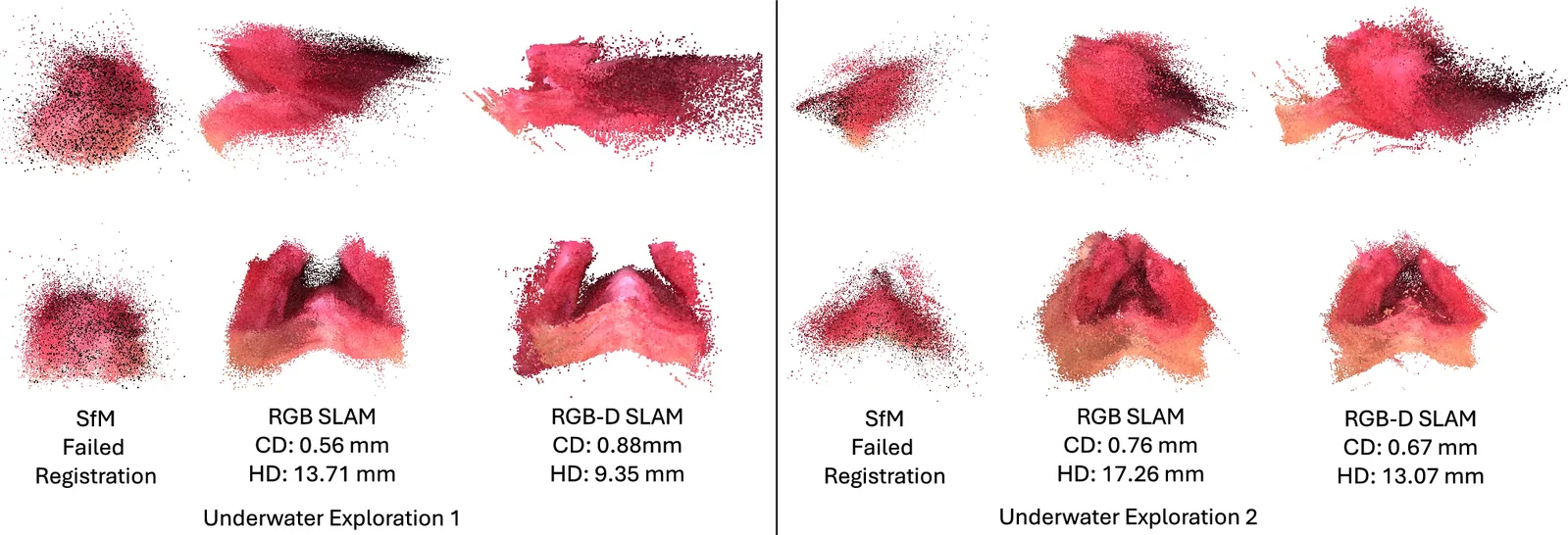
Reconstruction quality evaluation on submerged prostate phantoms. CD: One-sided Chamfer distance, HD: One-sided Hausdorff distance

To calculate the segmentation precision, we project the segmented tumor point cloud, that contains points from all of the images, back to the segmentation masks, using the estimated poses from reconstruction algorithm and the pinhole camera model. We analyze the successfully registered frames for SfM and keyframes for SLAM. The ratio of projected points that fall within the segmentation masks over the total gives us the precision of the segmentation projection. This reprojection process allows us to analyze the combined segmented points from different frames, and the effect of reconstruction algorithm through bundle adjustments, independently from the success of segmentation model. With this evaluation, we can see how the accumulation of masks, noise, and segmentation and reprojection errors affects projections from different viewpoints. We note that this evaluation method can be affected by projection of points associated with the areas occluded in the projected image; however, we evaluate all the methods consistently.

By changing the reconstruction parameters affecting the number of keyframes, point selection thresholds, and the number of features in the SfM pipeline, the balance between reconstruction speed and quality, or reconstruction density and noise can be changed. Finally, we note that coverage depends on the extent of exploration in the video and is only useful as a comparison between the methods.

**Fig. 6:**
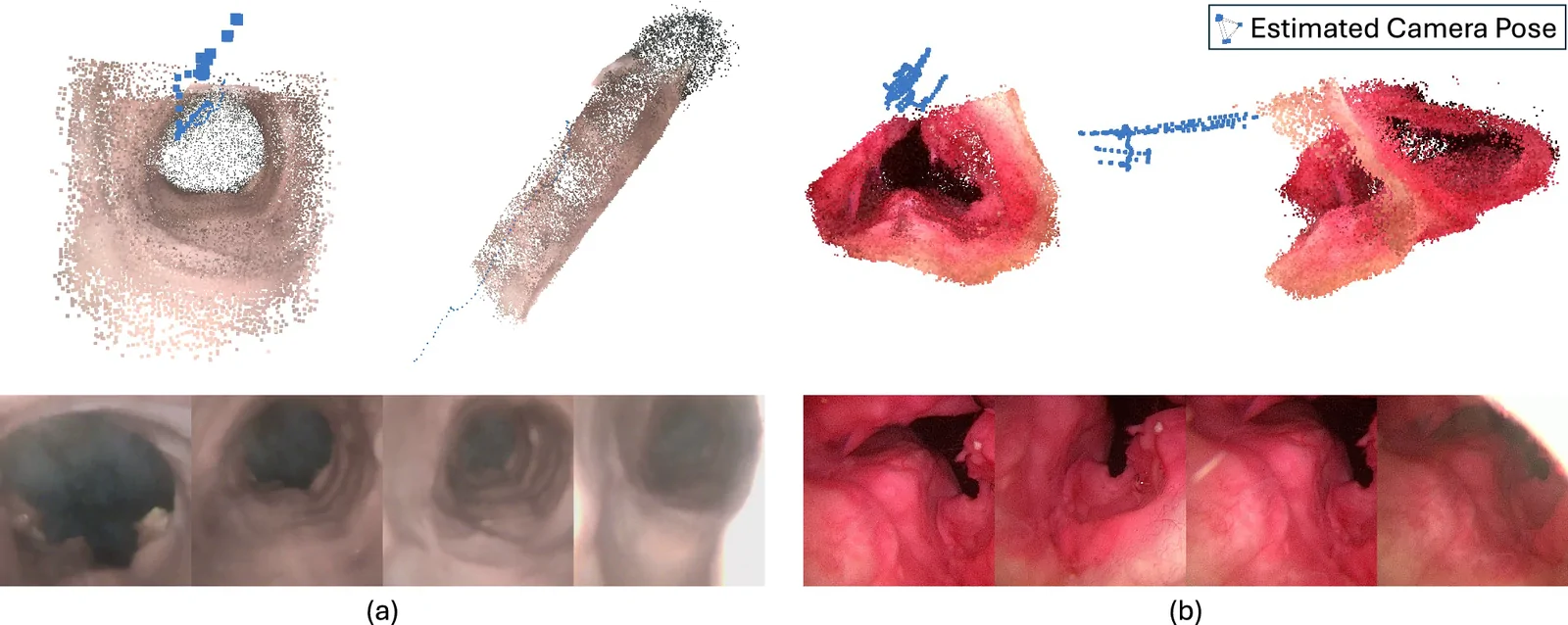
Qualitative examples showing reconstruction generalizability with RGB-D SLAM reconstructions and example frames from corresponding videos. **(a)** Reconstruction of a cadaver central airway. **(b)** Reconstruction of submerged prostate phantom, mid-operation, after one of the lobes is resected

Besides the comparative analysis in Table 1, we collected one operation and two exploration videos for the case when a prostate phantom is submerged underwater, and one cadaver central airway exploration video. These data are excluded from the comparative analysis due to a lack of registration visual cues in SfM reconstructions. Prostate exploration reconstruction analysis is given in Fig. 5, and qualitative results for cadaver and mid-operation reconstruction are given in Fig. 6.

### Registration accuracy

For registration and scaling evaluation, we collect one more exploration sequence with robot camera poses for each anatomy. To evaluate the accuracy of the registration, we calculate the root mean square error (RMSE) between robot-held camera poses and estimated registered camera poses from RGB-D SLAM algorithm. This residual error shows how well SLAM reconstructed the camera trajectory. To measure the accuracy of scaling, we cauterize small fiducial points in the anatomy and compare the real distances measured by calipers vs. estimated distances for 5 point pairs. Average of estimated distances over measured distances gives us the scale ratio, which would be 100% in the ideal scenario. We estimate a global scale factor for the reconstruction, which does not affect the reconstruction quality. We note that the accuracy of scaling and registration can be affected by the kinematics accuracy of the industrial arm holding the endoscope, and calibration quality.

## Discussion

From the quantitative results in Table 1, we see that the use of the SLAM algorithm results in quicker and more accurate reconstructions than the baseline SfM pipeline. The dense reconstruction nature of the SLAM algorithm, which relies on depth estimation, helps extend coverage to areas with a smaller number of observations and increases the number of reconstructed points. While both SLAM methods have higher reconstruction density and coverage compared to SfM algorithm, a decrease in average number of reconstruction points and coverage in the RGB-D SLAM compared to the RGB SLAM can be attributed to lower noise in the RGB-D reconstruction. This lower noise level can be understood better using the Hausdorff distance metric. However, real-time projection of segmentation masks without additional modification, such as weighting different masks as in [3], causes a lower segmentation precision. In both the CAO and the BPH cases, error metrics show similar trends.

**Table 2:** Evaluation of registration and scaling accuracy

	Scale ratio (%)	Pose RMSE (mm)
Central airway	98.17 ± 3.49	0.67
Prostate	98.23 ± 5.88	1.12

The performance of this pipeline is heavily dependent on its learning-based components. For the underwater scenario (Fig. 5), the depth model decreases the noise, leading to a smaller Hausdorff distance. However, since the underwater views are affected by the domain gap between images in air and water, the use of MDE results in larger Chamfer Distance and does not necessarily improve the reconstructions. Nonetheless, the overall pipeline can be easily adapted to different scenarios or procedures and shows a promising performance in cadaver experiment without any modifications (Fig. 6a). Although DROID-SLAM shows a good generalization for the endoscopic cases, with in-domain training of networks in the SLAM pipeline and better depth estimation models, the reconstruction qualities could be improved.

While SfM, with its offline nature, is more robust to temporary occlusions or other factors that can cause tracking to reinitialize, recovery in real time for SLAM is more challenging. This can also affect the segmentation precision and reconstruction accuracy. Even though the SLAM algorithm is able to reconstruct in the scenarios SfM failed (Fig. 5), modifications to real-time reconstruction pipeline may be needed in the clinical scenario where factors such as blood or debris are more apparent (Table 2).

Finally, as a limitation, this pipeline does not take the scene deformations into account and as the operation progresses, surgical scenes change significantly. Therefore, intermediate reconstructions can be used to update the scene representations (e.g., in Fig. 6b). This limitation is less apparent in CAO due to the rigid nature of the trachea and as can be seen in our previous work [3], surgical automation is still possible. Our future work includes improving the perception with deformation modeling and integration of other modalities, such as ultrasound. We plan to test the perception pipeline in downstream tasks and clinical videos.

## Conclusion

In this study, we presented a pipeline to create segmented 3D reconstructions for NOTES. These 3D maps can aid scene understanding and automation. We evaluated our pipeline by comparing with the ground-truth geometries obtained from CT scans and observed improvement in both processing times and reconstruction accuracies. Due to the plug-and-play nature of the pipeline and generalization capabilities of the used SLAM architecture, the methods presented in this paper can be extended to other anatomies or surgeries by simply changing the segmentation and depth estimation models, also making the overall proposed pipeline agnostic to domain shifts.

## Data Availability

Codebase for this project is available on GitHub: URL: github.com/vu-maple-lab/perseus

## Appendix 1

### Full pipeline evaluation results

**Table 3:** Average quantitative evaluation results for the RGB-D pipeline with automated scaling using pose registration

*Central airway obstruction*	
Median closest point dist. (mm) ↓	0.19 ± 0.07
One-sided Chamfer dist. (mm) ↓	0.31 ± 0.10
One-sided Hausdorff dist. (mm) ↓	4.76 ± 1.38
Segmentation precision (%) ↑	92.94 ± 6.99
# Reconstruct. points (x1000) ↑	249.8 ± 34.2
*Benign prostatic hyperplasia (Air/Submerged)*	
Median Closest point dist. (mm) ↓	0.40 / 0.78
One-sided Chamfer dist. (mm) ↓	0.64 / 1.08
One-sided Hausdorff dist. (mm) ↓	13.11 / 12.06
Segmentation precision (%) ↑	90.11 / 91.34
# Reconstruct. points (x1000) ↑	295.6 / 166.1

In the main Experiments and Results section, we evaluated reconstruction performance separately and compared it with alternative reconstruction approaches. This evaluation allows us to assess point cloud quality and geometric accuracy more directly, while reducing dependence on the robotic setup. Because our scaling and registration pipeline applies a global scale factor to the reconstructed scene, reconstruction quality directly affects the final registered point cloud.

This appendix complements the component-level analysis by evaluating the complete pipeline on a separately collected test dataset with the Virtuoso Surgical endoscopy system. Table 3 reports the final pipeline performance across two CAO cases, one air-filled BPH case, and one submerged BPH case. We note that camera control quality for exploration videos and the model deformation after the CT scans could affect the results. For the BPH experiments, the time between CT scanning and experiments is longer compared to the intervening time in the main section experiments, which may affect reconstruction evaluation as hydrogel phantoms shrink due to dehydration over time.
